# Supplementary material for: The genome sequence of the Silver-washed Fritillary, Argynnis paphia (Linnaeus, 1758) (Lepidoptera: Nymphalidae)
Source: Wellcome Open Res. 2025 Jul 31;10:399. [Version 1] doi: 10.12688/wellcomeopenres.24635.1 (PMC12426599; doi:10.12688/wellcomeopenres.24635.1)
Supplement: Peer review history

# Reviewer report: Reviewer response for version 1

**DOI:** 10.21956/wellcomeopenres.27149.r130579

**Published:** 2025-09-11

**Authors:** Jaakko Pohjoismäki

**Affiliations:** 1 University of Eastern Finland, Joensuu,, Finland

Yannick Chittaro, Andreas Sanchez, Kay Lucek, Charlotte J. Wright, Joana I. Meier, Mark L. Blaxter, the Tree of Life initiative, and the project Psyche community provide a genome report of *Argynnis paphia*. The genome meets Earth BioGenome Project (EBP) standards and represents a highly continuous, chromosome-level reference genome assembly with two assembled haplotypes.

 The introduction to the species is well written and informative, providing nicely also ecological context for the species. The sequencing, Hi-C–based chromosomal scaffolding, assembly, and annotation are carried out following the Darwin Tree of Life pipeline and appear technically sound and of high quality.

 I find little to improve in this report. One point of additional interest could have been a commentary on the two types of Z sex chromosomes, but this may be better addressed in a broader comparative framework with related species are available.

 Overall, this is a nice genome report and represents growing number of genomic resources for future research on Lepidoptera.

Are sufficient details of methods and materials provided to allow replication by others?

Yes

Is the rationale for creating the dataset(s) clearly described?

Yes

Are the datasets clearly presented in a useable and accessible format?

Yes

Are the protocols appropriate and is the work technically sound?

Yes

Reviewer Expertise:

molecular biology, biodiversity genetics, taxonomy

I confirm that I have read this submission and believe that I have an appropriate level of expertise to confirm that it is of an acceptable scientific standard.

# Reviewer report: Reviewer response for version 1

**DOI:** 10.21956/wellcomeopenres.27149.r130583

**Published:** 2025-09-03

**Authors:** Jason Charamis

**Affiliations:** 1 Foundation for Research and Technology - Hellas, Irákleion, Greece

This manuscript presents a reference genome for the silver-washed fritillary butterfly, *Argynnis paphia* . The work is technically sound and I have only a few text corrections to suggest:

 1. Instead of "Silver-washed Fritillary" write "silver-washed fritillary butterfly" in the first sentence of Background.

 2. Similarly in the second sentence of Background replace "Fritillary species" with "fritillary butterfly species"

 3. Correct this sentence and make the relevance of this study more clear

 "The reference genome presented here will allow to investigate the biogeography of this species and provide and invaluable resource for understanding the evolution of Lepidoptera."

Are sufficient details of methods and materials provided to allow replication by others?

Yes

Is the rationale for creating the dataset(s) clearly described?

Yes

Are the datasets clearly presented in a useable and accessible format?

Yes

Are the protocols appropriate and is the work technically sound?

Yes

Reviewer Expertise:

Arthropod Comparative Genomics

I confirm that I have read this submission and believe that I have an appropriate level of expertise to confirm that it is of an acceptable scientific standard.
